# Effects of Newcastle Disease Virus Infection on Chicken Intestinal Intraepithelial Natural Killer Cells

**DOI:** 10.3389/fimmu.2018.01386

**Published:** 2018-06-20

**Authors:** Mostafa Abdolmaleki, Swee Keong Yeap, Sheau Wei Tan, Dilan Amila Satharasinghe, Muhammad Bashir Bello, Mohammad Zareian Jahromi, Mohd Hair Bejo, Abdul Rahman Omar, Aini Ideris

**Affiliations:** ^1^Laboratory of Vaccines and Immunotherapeutic, Institute of Bioscience, Universiti Putra Malaysia, Serdang, Malaysia; ^2^China Asean College of Marine Science, Xiamen University Malaysia, Sepang, Malaysia; ^3^Faculty of Veterinary Medicine, Universiti Putra Malaysia, Serdang, Malaysia

**Keywords:** 28-4 IEL-NK cells, Newcastle disease virus, CD69, B-Lec, NK-LYSIN, B-NK, CHIR-AB1, IFN-γ

## Abstract

The intestinal intraepithelial natural killer cells (IEL-NK) are among the earliest effectors of antiviral immunity in chicken. Unfortunately, their role during Newcastle disease virus (NDV) infection remains obscure. Previous study has reported the development of a monoclonal antibody (mAb) known as 28-4, which is specifically directed against the CD3^−^ IEL-NK cells. In the present study, we used this mAb to investigate the effects of velogenic and lentogenic NDV infection on avian IEL-NK cells. Our findings revealed that chickens infected with velogenic NDV strains have a reduced population of purified CD3^−^/28-4^+^ IEL-NK cells as determined by flow cytometry. Furthermore, the CD3^−^/28-4^+^ IEL-NK cells from chicken infected with velogenic NDV strains were shown to have a downregulated expression of activating receptors (CD69 and B-Lec), effector peptide (NK-LYSIN), and IFN gamma. On the contrary, the expression of the inhibitory receptor (B-NK) and bifunctional receptor (CHIR-AB1) were upregulated on these purified CD3^−^/28-4^+^ IEL-NK cells following velogenic NDV infection. Meanwhile, the lentogenic NDV demonstrated insignificant effects on both the total population of CD3^−^/28-4^+^ IEL-NK cells and the expression of their surface receptors. In addition, using real-time PCR and transmission electron microscopy, we showed that CD3^−^/28-4^+^ IEL-NK cells were susceptible to velogenic but not lentogenic NDV infection. These findings put together demonstrate the ability of different strains of NDV to manipulate the activating and inhibitory receptors of CD3^−^/28-4^+^ IEL-NK cells following infection. Further studies are, however, required to ascertain the functional importance of these findings during virulent or avirulent NDV infection.

## Introduction

Newcastle disease (ND) is one of the most important viral diseases of birds that cause devastating economic losses in the global poultry industry. The disease is distributed worldwide and affects a wide range of avian species, causing high mortality and severe clinical symptoms (1). The etiologic agent of the disease is a negative-stranded RNA virus known as Newcastle disease virus (NDV), an avian paramyxovirus type 1, which belongs to the genus Avulavirus in the family *Paramyxoviridae* (2). Depending on the severity of clinical disease in chicken, NDV strains can be classified into three pathotypes: velogenic, mesogenic, and lentogenic strains. The velogenic strains are highly fatal (reaching 100% mortality) with clinical signs and lesions severely affecting the respiratory, gastrointestinal, and nervous system (3). On the other hand, the mesogenic strains are of intermediate virulence causing clinical illness in chickens characterized by moderate neurological and respiratory symptoms with low mortality. Meanwhile, the lentogenic strains such as LaSota, V4, and Ulster strains do not usually cause notable clinical disease in adult chickens and are used as live vaccines (4).

The innate immune system is the first line of defense against the invading microbial organisms. It is equipped with several molecular pathways that nonspecifically induce an antiviral state in the invaded host (5). Critical components of the innate immune barrier are natural killer cells (NK) which are cytotoxic lymphocytes capable of destroying transformed and virus-infected cells during the early phase of infection (6). They are also said to bridge the gap between the core innate defense mechanisms and the adaptive immune system by secreting cytokines, such as IFN-γ, IL-15, TNF-α, and IL-22 (7, 8). The biological functions of the NK cells are tightly regulated by two groups of receptors, the activating and inhibitory receptors, expressed on the surface of the NK cells. The inhibitory receptors recognize the major histocompatibility (MHC) molecules class I on the normal cells and prevent them from the cytotoxicity of the NK cells. Since MHC class I expression in altered cells, such as virus-infected cells, is either downregulated or missing, the inhibitory signals are abrogated, allowing the immediate killing of these altered cells by the NK cells (9). Similarly, the activating receptors are activated upon binding to certain stress ligands such as MHC complex class I chain A (MICA) expressed on the surface of virus-infected cells (10).

The role of chicken NK cells in the innate defense system was recognized more than two decades ago (11). Similar to human NK cells, avian NK cells have been characterized as large granular lymphocytes based on the morphologic features (12) and are known to express CD8aa homodimer without CD3 or immunoglobulin (CD3^−^/CD8^+^/TCR^−^) (12, 13). However, unlike the mammalian NK cells whose population is ~10% in the spleen and blood (14), the avian NK cells constitute less than 1% of peripheral blood lymphocytes or splenocytes in chicken (13). Interestingly, up to 50% of the total avian NK cells in chicken are found in the intestinal epithelium where they contribute to mucosal immune responses in the gut (15). With the development of a monoclonal antibody (mAb) known as 28-4, which specifically binds to CD3^−^ IEL-NK cell subsets located primarily in the chicken intestinal epithelium (13), more detailed immunological roles of these cells can be investigated.

Characterization of human or mouse activator and inhibitory NK receptors has been previously reported (16). Unfortunately, the expression of these receptors in avian NK cells during viral infections especially due to NDV is still poorly understood. Previous studies have shown that the NDV can deplete splenic T lymphocytes and increase the infiltration of macrophages (17), induce apoptosis of chicken IgM^+^ B cells (18), heterophils (19), and enhance the secretion of Th1-like proinflammatory cytokines (20). In addition, several viruses, such as avian influenza (21), human influenza (22), and chicken anemia virus (23), have evolved different mechanisms of escaping NK cell destructive activities. However, to the best of our knowledge, the manipulation of avian IEL-NK cells by NDV has not been previously examined. Therefore, in the present study, we investigated the immunological interactions of CD3^−^/28-4^+^ IEL-NK cells with velogenic and lentogenic strains of NDV. Our findings revealed that velogenic, but not lentogenic NDV, downregulates the activating receptors on chicken CD3^−^/28-4^+^ IEL-NK cells, and in turn increase the expression of the inhibitory receptors. We also demonstrated a substantial depletion of CD3^−^/28-4^+^ IEL-NK cell population following infection with velogenic NDV in chicken. In contrast, lentogenic NDV infection produced only a slight effect on the total population of CD3^−^/28-4^+^ IEL-NK cells. Our results, therefore, highlight the differential modulation of avian IEL-NK cells following velogenic and lentogenic NDV infections. This is the first study on the molecular interactions of NDV with intestinal CD3^−^/28-4^+^ IEL-NK cells in chicken.

## Materials and Methods

### Chickens and Viruses

Eight-day-old specific-pathogen-free (SPF) chicken embryonated eggs were obtained from Malaysian Vaccines and Pharmaceuticals and hatched in our laboratory. Chickens were transferred to the BSL2 facility and housed in isolators where they were fed on commercial feed and drank water *ad libitum*. Two velogenic (AF2240-I and IBS005/11) and one lentogenic (LaSota) strains of NDV were propagated, titrated, aliquoted, and stored at −80°C until needed for experimental challenge. To propagate the viruses, 9-day-old SPF embryonated eggs were inoculated with 0.1 ml of the diluted viruses and incubated at 37°C for 2–4 days. Eggs with dead embryos were immediately chilled overnight to harvest the infected allantoic fluid. Hemagglutination/hemagglutination inhibition tests were performed following virus propagation. The titer of each virus expressed as mean egg lethal dose (ELD_50_) or mean egg infectious dose (EID_50_) was determined using Reed and Muench method. On the day of the challenge, aliquots of the viruses were diluted in sterile PBS to 10^6^ ELD_50_/ml.

### Experimental Chicken Trial

Immediately before the challenge experiment, three 21-day-old chicken were randomly selected and sacrificed to serve as uninfected controls. The remaining chickens were divided into three groups comprising 18 birds each. Groups I, II, and III were inoculated with 0.1 ml of 10^6^ ELD_50_/ml of AF2240-I, IBS005/11, and LaSota, respectively, *via* the oculonasal route. From each of these infected groups, six chickens were humanely sacrificed to collect their duodenum at 12, 36, and 72 hours post infection (hpi), respectively. The trial was independently repeated three times. Duodenum samples obtained from the uninfected birds and the infected groups were collected in cold Roswell Park Memorial Institute medium reagent (RPMI) (Sigma Aldrich, USA) for further analysis.

For the bioassay including quantitative real-time PCR and flow cytometry, the six chickens collected at each time point per group were pooled two chickens as one and resulted in three biological replicates for each time point per group. Three technical replicates were performed on each biological replicates.

### Isolation of Chicken Intraepithelial Lymphocyte (IEL) Cells

Isolation of IEL was performed as previously described with slight modification (15). Briefly segments of the duodenum (about 7 cm) were opened longitudinally and cut into roughly 2-cm fragments. To remove the intestinal mucus, the cut tissue fragments were initially washed with fresh RPMI followed by pre-warmed PBS/EDTA/DL-Dithiothreitol (DTT) (Sigma-Aldrich, USA) solution and then incubated in a shaking water bath at 37°C for 30 min. The samples were later homogenized using a 70-µM cell strainer (SPL Life Science, Korea). Dead cells from the single cell suspension were discarded following Histopaque-1119 (Sigma Aldrich, USA) density gradient centrifugation and the cell viability was determined using Trypan blue dye exclusion method and Scepter 2.0. Finally, the cells were divided into two parts, one part was stained for immunophenotyping of IEL and the second part was processed by magnetic activated cell sorting (MACS) (Miltenyi Biotec, Germany) for selective characterization.

### Isolation of CD3^−^ IEL Cells

To separate the CD3^−^ cells from other IEL, we employed MACS based cell separation technology. Accordingly, a total of 10^8^ isolated IEL cells/ml were washed with cold PBS/BSA/EDTA at 4°C for 10 min at 400 × *g*. The pellet was then re-suspended in 100 μl cold PBS/BSA/EDTA and 10 μl CD3 PE (Southern Biotech, USA) and mixed by vortexing. The cells were later incubated on ice and shaken at 100 rpm for 30 min. Then, 1 ml of cold PBS/BSA/EDTA was added and centrifuged at 4°C for 10 min. After that, 100 μl cold PBS/BSA/EDTA and 10 μl Anti PE Micro Bead (Miltenyi Biotec, Germany) were added, vortexed, and incubated on ice for 30 min. The cells were washed again and re-suspended in 1 ml cold PBS/BSA/EDTA. Finally, the MACS column was prepared and, the columns were rinsed with cold PBS/BSA/EDTA and the cells were applied to the columns in a magnetic field. All CD3^−^ cells passed from the columns were collected and put on ice for further characterization.

### Enrichment of CD3^−^/28-4^+^ IEL-NK Cells

A total of 10^8^ CD3^−^ cells/ml were re-suspended by 100 μl cold PBS/BSA/EDTA and 30 μl 28-4 Mab, vortexed, and incubated on ice for 30 min followed by shaking at 100 rpm in dark foil. The 28-4 mAb was kindly provided by Prof. Thomas Göbel, Ludwig-Maximilians-Universität München, Germany. Unless stated otherwise, all subsequent shaking incubations were carried in the dark and at 100 rpm while the centrifugation steps were performed at 400 × *g* for 10 min at 4°C. Accordingly, the cells were washed and centrifuged before the supernatant was removed and replaced with 100 μl cold PBS/BSA/EDTA and 12 μl Goat-Anti-Mouse-IgG3 (Abcam, USA). The cells were then re-suspended by vortexing, incubated on ice, and shaken as described above. Next, the cells were washed with 100 μl cold PBS/BSA/EDTA, stained with 10 μl Anti FITC Micro Bead (Miltenyi Biotec, Germany), and again shaken on ice as previously described. Subsequently, the MACS column was prepared and the columns were rinsed with cold PBS/BSA/EDTA prior to the application of cells suspension in a magnetic field. The 28-4^+^ cells bound to the column while other cells passed out. Later, the column was rinsed with cold PBS/BSA/EDTA three times and then removed from the magnet. Finally, the 28-4^+^ cells were eluted, counted, and aliquoted into three tubes for further characterization such as immunophenotyping, transmission electron microscopy (TEM), and quantitative gene expression assay.

### Flow Cytometric Analysis of IEL-NK Cells

To determine the subpopulation of 28-4^+^ NK cells in IEL cells from the duodenum tissue of control and NDV infected chickens, the isolated IEL cells were stained with 28-4 mAb. Briefly, 2 million cells/ml were re-suspended in 100 μl cold PBS mixed with 10 μl 28-4 Mab, incubated, and shaken on ice for 30 min. After centrifugation, the supernatant was removed and 100 μl cold PBS mixed with 12 μl Goat-Anti-Mouse-IgG3 (Abcam, USA) and then incubated with shaking on ice for 1 h. Furthermore, to check the purity of isolated CD3^−^/28-4^+^ IEL-NK cells, the cells were co-stained as described in the enrichment section. Cells were then washed and, 2 ml of 1% (v/v) buffered formalin (Sigma, Aldrich, USA) was mixed with the stained cells for immunophenotyping and kept at 4°C in dark foil. FACSCalibur™ flow cytometer (BD Biosciences, USA) and CellQuest Pro™ software (BD Biosciences, USA) were used in analyzing the results.

### Electron Microscopy of CD3^−^/28-4^+^ IEL-NK Cells

Purified CD3^−^/28-4^+^ IEL-NK cells were fixed using 2.5% Glutaraldehyde (Sigma Aldrich, USA) for 4–6 h. The cells were then submerged in proper animal serum and later sliced into 1 mm^3^ before another fixation in 2.5% Glutaraldehyde at 4°C for 1–2 h. Next, the cells were washed three times with 0.1 M of sodium Cacodylate Buffer (Sigma Aldrich, USA) for 10 min at 400 × g followed by another fixation with 1% Osmium Tetroxide (Sigma Aldrich, USA) at 4°C for 2 h. The cells were then dehydrated for 10 min each in 35, 50, 75, and 95% acetone percentages and 15 min in 100% acetone. The dehydrated samples were placed in a full resin beam capsule and polymerized in the oven at 60°C for 24–48 h. Later, the samples were cut to 1 μm, stained with Toluidine Blue (Sigma-Aldrich, USA), and then the area of interest was examined using a light microscope. Finally, ultrathin sectioning was performed before the cells were stained with uranyl acetate for 15 min and viewed under the Electron Microscope (TEM) (Hitachi H-7100, Japan).

### Quantitative Real-Time Reverse Transcription PCR (RT-qPCR)

At different time points post infection, CD3^−^/28-4^+^ IEL-NK cells were collected to estimate the viral copy numbers using RT-qPCR. Prior to RNA extraction, samples from all the groups were standardized to contain five million cells using the Scepter 2.0 for all groups. The extracted RNA was used for absolute quantification of the viral load using TaqMan real-time PCR assay (17). The following primers and probe targeting the F gene of velogenic strains were used: Forward primer 5′ TCCGCAAGATCCAAGGGTCT 3′, Reverse primer 5′ CGCTGTTGCAACCCCAAG 3′ and Probe 5′ (6FAM)-AAGCGTTTC TGTCTCCTTCCTCCA-(BHQ1) 3′. In the case of chicken infected with lentogenic NDV (LaSota) strain, the primers used were: forward primer 5′ TCCGTAGGATACAAGAGTCT 3′, reverse primer 5′ GGCAGTTGCAACCCCAAG 3′ and 5′ (HEX) CCTATAAGGCGCCCCTGTCTCCC (BHQ) 3′ served as the probe. The concentration of the extracted RNAs was standardized and then subjected to one–step RT-qPCR using iTaq Universal Probes One-Step Kit (BioRad, USA) according to the manufacturer’s protocol. The cycling program consists of 50°C for 10 min, 95°C for 3 min, followed by 40 cycles of 95°C for 20 s, 58°C for 30 s, and finally 72°C for 15 s. The amplification of the positive control RNA was performed by 10-fold serial dilution (from 1,000 to 0.01 ng/µl per reaction) and each dilution was prepared in triplicate in a single run.

Total NK cellular RNA was extracted from samples and collected at the time points 0, 12, 36, and 72 hpi using Master Pure™ RNA purification kit (Epicentre, USA). The RNA was reverse transcribed using a high capacity RNA-to-cDNA kit (Applied Biosystems, USA) according to the manufacturer’s protocol and subjected to quantitative real-time PCR using BioRad CFX-96 system (BioRad, USA). A total of five NK genes, namely, CD69 and B-Lec (activators), B-NK (inhibitor) CHIR-AB1 (bifunctional), NK-LYSIN (effector peptide), and IFN-γ (Th1-like cytokine) were selected to estimate their relative mRNA expression in both the NDV infected and non-infected samples. Three housekeeping genes namely, glyceraldehyde-3-phosphate-dehydrogenase, 18S ribosomal RNA, and beta-actin were used for normalization of gene expression. The sequences of primers and probes used in this experiment are found in Table S1 in Supplementary Material. Quantification of fold change expressions of NK cell-related genes were carried out using CFX Manager software version 3.1 (BioRad, USA).

### Indirect Immunofluorescence Staining (IF)

The paraffin-embedded sections of the duodenum samples were mounted on polysine slides (Thermo Scientific, Germany) and processed for immunohistochemistry staining. Briefly, the samples were subjected to deparaffinization and a series of rehydration steps with ethanol before permeabilization with 0.3% Triton X-100 (Sigma-Aldrich, USA). After washing steps, the antigen was retrieved using Heat-Induced Epitope Retrieval method. Next, the washed samples were followed by blockage of non-specific binding sites using 5% Normal Goat Serum (Abcam, USA). The slide was then incubated at 4°C overnight in 1:100 dilution of 28-4 Mab followed by staining with FITC-conjugated Goat anti-Mouse IgG3 secondary antibody (Abcam, USA) at a dilution of (1:100) in the wet and dark chamber for 1 h at room temperature. Slides were washed and mounted with anti-fade mounting media (Abcam, USA) and then observed under a confocal microscope (Zeiss, Germany).

### Statistical Analysis

Mean ± SD was used to present the results. Two-way ANOVA followed by the Tukey’s test was carried out to show the differences between means where *p* ≤ 0.05 was selected as statistically significant.

## Results

### Reduction of IEL and CD3^−^/28-4^+^ IEL-NK Cells in Chicken Infected With Velogenic NDV

To determine the effect of different NDV pathotypes on the population of IEL, the cells were counted before the infection and at different time points post infection. A total number of 220 × 10^6^ viable IEL cells were estimated in the uninfected control birds. After inoculation with the two velogenic NDV strains, the population of IEL cells showed a decreasing trend as the infection progressed. Chicken infected with the NDV IBS005/11 strain demonstrated the highest IEL reduction compared to those infected with AF2240-I strain. On the contrary, LaSota inoculated chickens only showed a slight reduction in the total number of IEL cells even after 72 hpi. Furthermore, among the total viable IEL obtained from the uninfected chicken, 70 × 10^6^ were CD3^−^/28-4^+^ IEL-NK cells. However, the population of these cells was also consistently reduced as the infection with velogenic strains progressed. On the other hand, chickens inoculated with LaSota strain did not produce a significant reduction in the number of CD3^−^/28-4^+^ IEL-NK cells at different hours post infection (Table 1).

**Table 1:** Cell count.

NDV strain	Cells	Number of natural killer cells (NK) at different hours post infection (hpi)		
		12	36	72
AF2240-I	IEL	120 × 10^6^ ± 0.72a,1*	64 × 10^6^ ± 0.47^a,2^*	47 × 10^6^ ± 0.47^a,3^*
	CD3^−^/28-4^+^ IEL-NK	15 × 10^6^ ± 0.47d,4*	13.5 × 10^6^ ± 0.36^5^*	11 × 10^6^ ± 0.47^d,7^*
IBS005/11	IEL	59 × 10^6^ ± 0.47^b,1^*	45 × 10^6^ ± 0.72^b,2^*	8.6 × 10^6^ ± 0.14^b,3^*
	CD3^−^/28-4^+^ IEL-NK	36.5 × 10^6^ ± 0.49^e,4^*	13.5 × 10^6^ ± 0.36^e,6^*	NA
LaSota	IEL	130 × 10^6^ ± 1.19^c,1^*	135 × 10^6^ ± 0.47^c,2^*	141 × 10^6^ ± 0.72^c,3^*
	CD3^−^/28-4^+^ IEL-NK	61 × 10^6^ ± 0.47^f,4^*	47 × 10^6^ ± 0.47^f,5,6^*	39 × 10^6^ ± 0.47^f,7^*

*NA, not available*.

*Numbers of uninfected control intraepithelial lymphocyte (IEL) cells (220 × 10^6^ ± 1.19 cells) and uninfected control CD3^−^/28-4^+^ IEL-NK cells (70 × 10^6^ ± 0.47 cells). Values with asterisk compared to control differ significantly. Values with same letters differ significant between different NDV strains. Values with same number differ significant between different time points by using two-way ANOVA (*p* ≤ 0.05). The value was the means ± SEM of three experiments*.

*Values with same letter differ significantly between different NDV strains*.

*Values with same number differ significantly between different time points*.

### Immunofluorescence Detection of 28-4^+^ IEL-NK Cells

**Figure 1:**
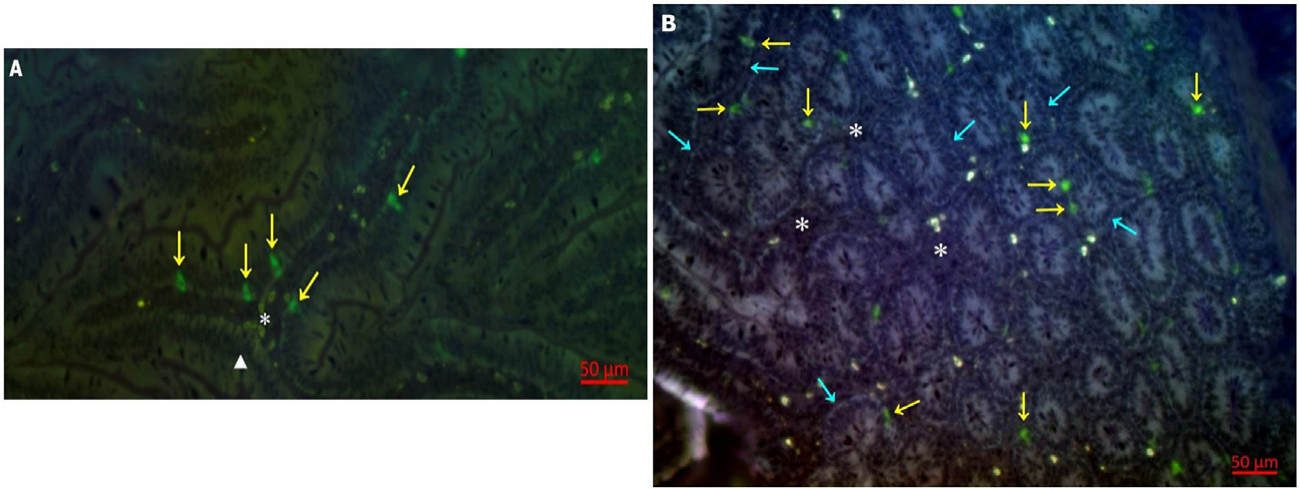
Distribution of 28-4^+^ IEL-NK cells in duodenum of uninfected control chickens. **(A)** natural killer cells (NK) (yellow arrow), villi epithelium (triangle), lamina propria (asterisk). **(B)** Intestinal glands (blue arrow), NK cell (yellow arrow), lamina propria (asterisk).

Having determined the proportion of 28-4^+^ IEL-NK cells in the duodenum tissues of chicken, we attempted to determine the distribution and location of the cells in the duodenum using indirect immunofluorescence assay. Our results demonstrated that 28-4 positive NK cells are preferentially localized primarily at the villi epithelium (Figure 1A) and intestinal glands (Figure 1B).

### TEM Examination of CD3^−^/28-4^+^ IEL-NK Cells

**Figure 2:**
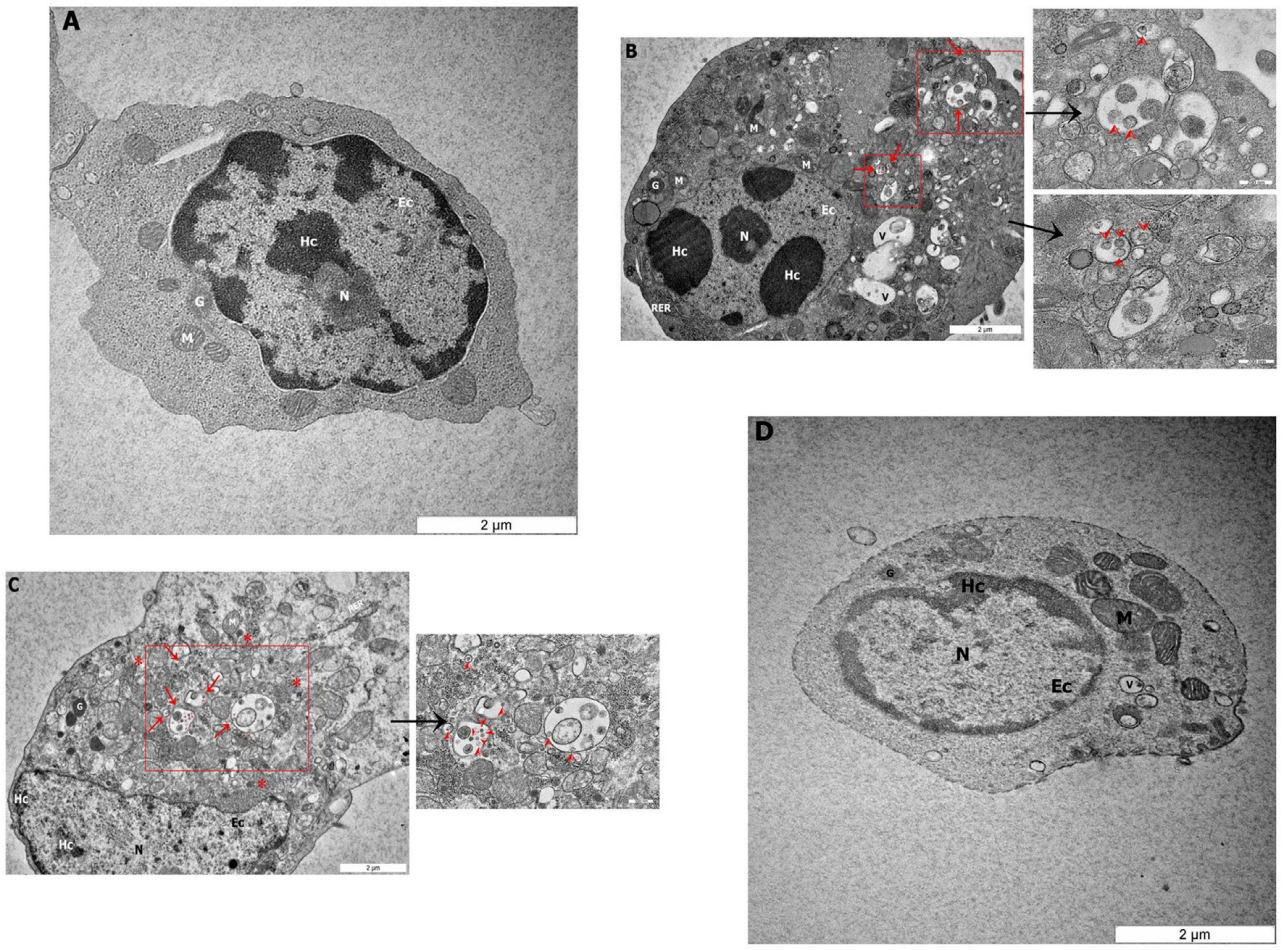
Transmission electron microscopy micrograph of CD3^−^/28-4^+^ enriched IEL-NK cells from duodenal loop of uninfected and infected chickens. The picture demonstrated **(A)** the uninfected (control) natural killer cells (NK) with normal structures. The NK cell isolated from duodenum infected with velogenic groups, AF2240-I **(B)**, IBS005/11 **(C)** and lentogenic group, LaSota **(D)**. **(B,C)** Variable amounts of viral particles are taken up into vesicular structures (red arrow). The smaller boxes show the higher resolution of the viral particles. **(D)** The picture demonstrated the LaSota NK cell with less moderate mitochondria and the chromatin was lower condensed compared to control. Organelle marks: mitochondria (M), granule (G), rough endoplasmic reticulum (RER), nucleus (N), heterochromatin (Hc), euchromatin (Ec), vesicular structures (red arrow), viral particles (red arrowhead), vacuole (V), ribosomal aggregation (red asterisk).

To determine if NDV can infect avian CD3^−^/28-4^+^ IEL-NK cells, ultrathin sections of the duodenum from control and infected chickens were examined by TEM. Isolated CD3^−^/28-4^+^ IEL-NK cells from uninfected control chickens showed a normal distribution of organelles. In particular, the cells showed a granular and abundant cytoplasm containing a large nucleus, a number of ribosomes, and other organelles (Figure 2A). On the contrary, the ultrastructure of CD3^−^/28-4^+^ IEL-NK cells isolated from chicken infected with velogenic NDV showed remarkable alterations in the morphology and composition of the cells. Notably, distinct virus-like particles were detected within the cytoplasm of the infected cells (Figures 2B,C). In addition, abundant fragmented and irregularly shaped mitochondria and many vacuolizations and compaction of cytoplasmic organelles were observed. Furthermore, the amount of heterochromatin decreased compared with CD3^−^/28-4^+^ IEL-NK cells from the control group. However, the CD3^−^/28-4^+^ enriched IEL-NK cells obtained from chicken infected with lentogenic NDV were shown to be ultra-structurally nearly normal. There were moderate numbers of mitochondria in the cytoplasm as well as less nucleus chromatin compared to uninfected control chickens. No virus particle was detected in the CD3^−^/28-4^+^ IEL-NK cells from chicken inoculated with LaSota NDV strain (Figure 2D).

### Real-Time PCR Quantification of NDV in CD3^−^/28-4^+^ IEL-NK Cells

To determine the quantity of both velogenic and lentogenic NDV in CD3^−^/28-4^+^ IEL-NK cells, viral load (mRNA level) was determined in the infected chicken by qRT-PCR at 0, 12, 36, and 72 hpi. No virus RNA was detectable in CD3^−^/28-4^+^ IEL-NK cells collected from the uninfected control and LaSota infected groups at various time points post infection. Similarly, at 12 hpi viral RNA was undetectable among chicken infected with both velogenic strains (AF2240-I and IBS005/11). The two velogenic strains were first detected at 36 hpi after which further increase in the viral copy number was noted for AF2240-I at 72 hpi. However, viral copy number evaluation was not performed at 72 hpi for chicken infected with IBS005/11 due to a low number of CD3^−^/28-4^+^ IEL-NK cells (Table 2).

**Table 2:** Detection of Newcastle disease virus (NDV) copy number from CD3^−^/28-4^+^ enriched IEL-NK cells.

NDV		Hours post infection (hpi)	Viral copy number (log10)
Strain	Genotype		
AF2240-I IBS005/11 LaSota	VII VIII II	0	ND ND ND
AF2240-I IBS005/11 LaSota	VII VIII II	12	ND ND ND
AF2240-I IBS005/11 LaSota	VII VIII II	36	7.06 ± 0.01 7.22 ± 0.07 ND
AF2240-I IBS005/11 LaSota	VII VIII II	72	7.95 ± 0.51 NA ND

*ND, not detected; NA, not available*.

*Detection of viral copy number from CD3^−^/28-4^+^ enriched IEL-NK cells of specific-pathogen-free chickens inoculated with different NDV strains using Taqman quantitative real-time PCR. The value was the means ± SEM of three experiments*.

### Immunophenotyping of IEL-NK Cells

**Figure 3:**
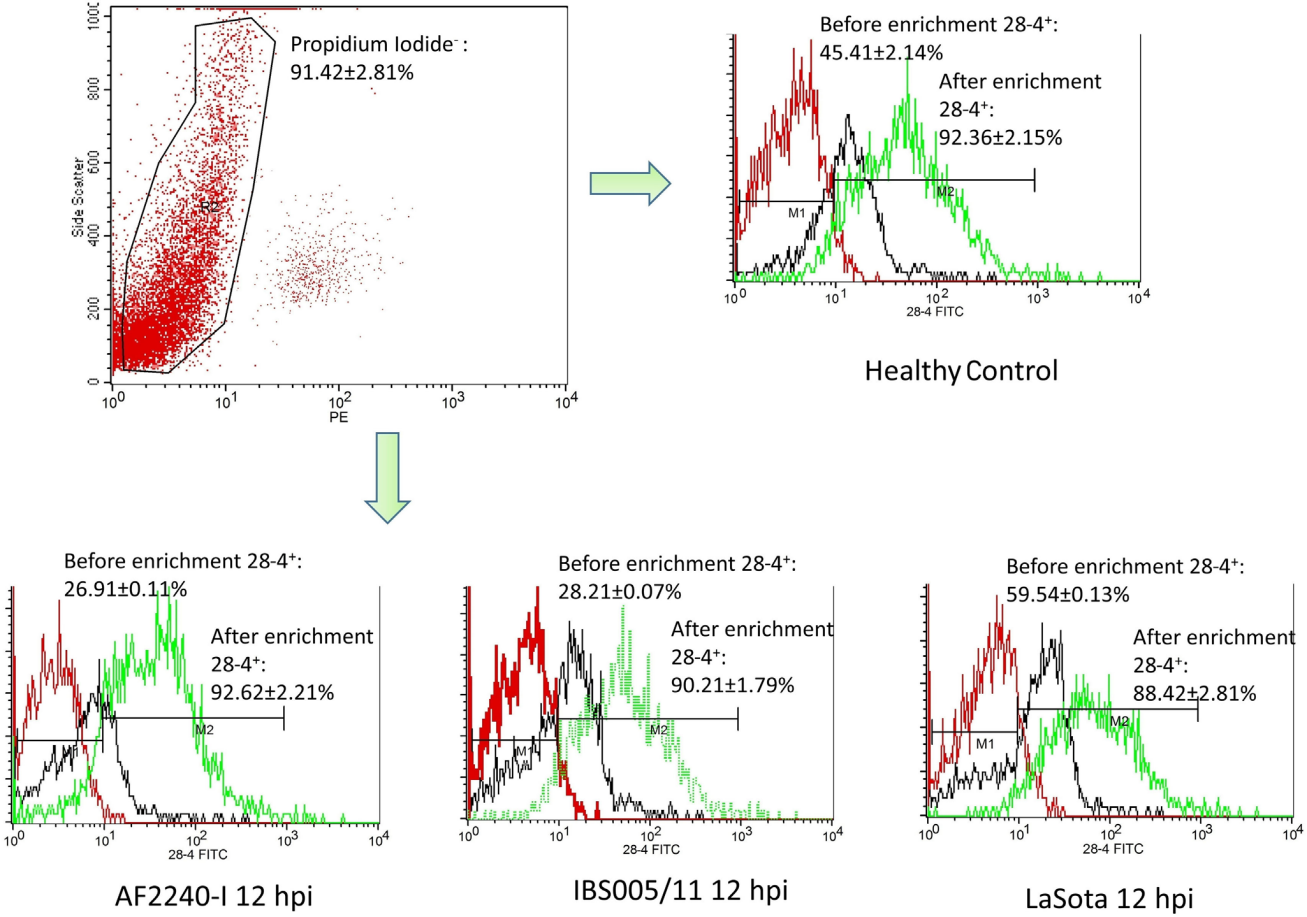
Flow cytometry histogram profile for isotype control (red line); before enrichment (black line) and after enrichment (green line) for 28-4^+^ IEL-NK cells from uninfected control chickens (day 0) and Newcastle disease virus challenged (after 12 hpi of AF2240-I, IBS005/11, and LaSota). The histogram was gated based on propidium iodide population that representing ~90% of the total population of IEL cells. The value was the means ± SEM of three experiments.

The optimized method of enriching the IEL consistently yielded a high purity of CD3^−^/28-4^+^ cells of more than 92%. The percentage of 28-4 positive cells in the IEL population harvested from control chickens was 45% (Figure 3). However, the proportion of these varied with the time point post exposure and the virus strain used. The highest percentage of 28-4^+^ IEL-NK cells was detected at 12 hpi with different NDV strains. However, as the infection progressed, the percentage of these cells gradually declined with a significantly higher reduction in the percentage of the cells recorded from the velogenic NDV infected chickens (Table 3). In addition, the flow cytometry detection of 28-4 was couple with propidium iodide as first selection. We have used IgG3 isotype control for gating of 28-4^+^ cells. Dead cells (which is ~10%) was excluded based on the SSC + propidium iodide gating (Figure 3).

**Table 3:** Percentage of 28-4^+^ cells in total IEL-NK cells obtained from chickens infected with different Newcastle disease virus (NDV) strains.

NDV strain	28-4^+^ IEL-NK cells		
	12 hpi	36 hpi	72 hpi
AF2240-I	26.91 ± 0.11^a,1^*	25.90 ± 0.55^b,3^*	5.66 ± 0.24^a,b,4^*
IBS005/11	28.21 ± 0.07^c,2^*	23.71 ± 0.16^c,3^*	10.18 ± 0.13^c,4^*
LaSota	59.54 ± 0.13^d,1,2^*	57.47 ± 0.11^d,3^*	41.22 ± 0.92^d,4^*

*For uninfected control, the percentage for 28-4^+^ cells was 45.41 ± 2.14. Values with asterisk compared to control differ significantly. Values with same letters differ significant between different NDV strains. Values with same number differ significant between different time points by using two-way ANOVA (*p* ≤ 0.05). The value was the means ± SEM of three experiments*.

*Values with same letter differ significantly between different NDV strains*.

*Values with same number differ significantly between different time points*.

### Relative Fold Change Expression Profiles of Selected NK Cell-Related Genes and Cytokine

The expression profiles of two activator receptors (CD69, B-Lec), one inhibitory receptor (B-NK), one bifunctional NK cell receptor (CHIR-AB1), an effector peptide (NK-LYSIN) and a T-helper 1-like cytokine (IFN-γ) were evaluated following NDV infection in chickens. The relative fold changes of these genes in CD3^−^/28-4^+^ IEL-NK cells from SPF chickens infected with different NDV strains were compared to the uninfected control chickens at 12, 36, and 72 hpi. Interestingly, in chicken infected with velogenic strains, activator receptors, effector peptide, and IFN-γ genes were all downregulated at 12–72 hpi with the highest down-modulation of CD69 (eightfold) in chickens infected with IBS005/11 strain while the peak level of B-Lec downregulation (fivefold) was observed at 72 hpi in the AFF2240-I infected chickens. In addition, about threefold and fourfold decreased expressions of both IFN gamma and NK-LYSIN were detected in the AF2240-I infected group, respectively. On the contrary, the inhibitory NK gene showed an opposite pattern with AF2240-I infected group causing a threefold upregulation of the B-NK gene at 72 hpi. Similarly, the expression of CHIR-AB1 was increased (22-fold) in chickens infected with AF2240-I strain. However, in the case of the LaSota infected group, the gene upregulation is marginal as compared to other groups (Table 4).

**Table 4:** Relative folds change expression profiles of immune-related genes associated with NK cell activity of CD3^−^/28-4^+^ IEL-NK cells following Newcastle disease virus (NDV) infection.

NDV strain	Immune-related gene	Expression profiles in CD3^−^/28-4^+^ IEL-NK cells at different hours post infection (hpi)		
		12	36	72
AF2240-I	CD69	−1.28 ± 0.07^a,1^*	−2.13 ± 0.07^b,3^*	−2.6 ± 0.05^a,b,4^*
	B-Lec	−2.01 ± 0.17^a,1^*	−3.37 ± 0.41^b,3^*	−5.4 ± 0.45^a,b,5^*
	B-NK	1.92 ± 0.16^a^	2.33 ± 0.32^b^*	3.09 ± 0.11^a,b,1^*
	CHIR-AB1	22.02 ± 0.07^a,1^*	7.98 ± 0.06^a,2^*	4.11 ± 0.19^a,3^
	NK-LYSIN	−1.03 ± 0.08^a,1^*	−2.49 ± 0.15^b,3^*	−4.34 ± 0.06^a,b,5^*
	IFN-γ	−1.14 ± 0.1^a,1^	−1.39 ± 0.09^b,3^	−3.78 ± 0.01^a,b,5^*

IBS005/11	CD69	−2.04 ± 0.34^c,2^*	−8.05 ± 0.23^c,3^*	NA
	B-Lec	−1.17 ± 0.19^2^	−2.01 ± 0.19^4^*	NA
	B-NK	1.48 ± 0.05	2.18 ± 0.26	NA
	CHIR-AB1	5.74 ± 0.12^1^*	4.88 ± 0.07^2^*	NA
	NK-LYSIN	−1.21 ± 0.05^2^*	−1.42 ± 0.05^4^*	NA
	IFN-γ	−3.63 ± 0.23^1,2^*	−2.3 ± 0.2^3,4^*	NA

LaSota	CD69	1.17 ± 0.36^1,2^	1.58 ± 0.1^3^	1.24 ± 0.23^4^
	B-Lec	1.24 ± 0.2^1,2^	1.56 ± 0.57^3,4^	1.38 ± 0.05^5^
	B-NK	1.09 ± 0.01	1.98 ± 0.06	1.33 ± 0.08^1^
	CHIR-AB1	1.47 ± 0.27^1^	1.26 ± 0.51^2^	1.14 ± 0.32^3^
	NK-LYSIN	1.47 ± 0.31^1,2^	1.88 ± 0.24^3,4^	1.3 ± 0.17^5^
	IFN-γ	1.26 ± 0.07^2^	1.02 ± 0.07^4^	1.05 ± 0.53^5^

*NA, not available*.

*Values with asterisk compared to control differ significant. Values with same letters differ significant between different NDV strains. Values with same number differ significantly between different time points. Analyzing was carried out using two-way ANOVA (*p* ≤ 0.05). The value is the means ± SEM of three experiments*.

*Values with same letter differ significantly between different NDV strains*.

*Values with same number differ significantly between different time points*.

## Discussion

Mucosal surfaces in the urogenital, gastrointestinal, and respiratory systems are the major ports of entry for most viral pathogens (24). They are well armed with immune cells and other complex immunological mechanisms that ultimately limit virus spread and multiplication in the infected hosts. Among the key players in the mucosal immunity are the IEL-NK cells, whose cytotoxicity and interferon gamma production ensures early defense against the invading viral pathogens (25). However, viruses have evolved several mechanisms to evade NK cell-mediated antiviral response. For instance, highly pathogenic avian influenza virus has recently been reported to decrease the activation of lung NK cells in order to enhance its spread in avian hosts (21). In another study, avian influenza virus was shown to dodge the antiviral activity of NK cells by directly attacking and inducing apoptosis in the cells (26). Furthermore, velogenic NDV has been shown to attack many immune cells in the chicken gut and other organs, as part of the mechanisms to dodge the host’s immune response. Unfortunately to date, the interaction of NDV with the CD3^−^/28-4^+^ IEL-NK cells located in the chicken gut is yet to be clearly elucidated. Therefore, we report herein for the first time, the effects of different NDV pathotypes on the expression profiles of the activating and inhibitory receptors of avian CD3^−^/28-4^+^ IEL-NK cells.

Based on IF, we demonstrated that the 28-4^+^ IEL-NK cells are randomly distributed in the villi epithelium (Figure 1A) and intestinal glands (Figure 1B) found in the chicken duodenum, as previously reported (27). Flow cytometric evaluation using the 28-4 mAb developed by Göbel and co-workers (13) also revealed that, more than 45% of the total IELs in chicken duodenum represent 28-4^+^ NK cells (Figure 3). To determine if NDV affects the population of 28-4^+^ cells within the IEL cells, we further analyzed the cells’ proportion at various time points following velogenic and lentogenic NDV infection (Table 3). Our findings revealed that, although a reduction in the total number of IELs or the 28-4^+^ cells within the IELs was noticed following infection with both NDV pathotypes, chickens infected with the velogenic NDV showed a more pronounced reduction of both cell types compared to those infected with the lentogenic strain. Interestingly, this finding correlates with the viral load result which indicates an increase in the copy number of velogenic but not lentogenic strains of NDV during the course of infection (Table 2). It is, however, important to mention that the observed reduction in the total IELs might not specifically be due to the reduced number of 28-4^+^ cells alone. Reduction in other constituents of the IELs such as T and B cells might have contributed to the present finding. Indeed, velogenic NDV has previously been shown to reduce the population of T cells (17) and induce apoptosis of B lymphocytes (18) in the infected chickens. Nevertheless, the marked reduction in the population of the purified CD3^−^/28-4^+^ IEL-NK cells following velogenic NDV infection is an indication that the cells are considerably affected by the virus. Hence, it can be speculated that the pathogenicity of the velogenic NDV in chicken GIT might be associated with its ability to depopulate the intestinal CD3^−^/28-4^+^ IEL-NK cells.

To explore the specific effects of NDV on the CD3^−^/28-4^+^ IEL-NK cells, we examined before and after NDV infection, the expression profiles of receptors that control the immunological functions of the NK cells. Interestingly, we found that velogenic and lentogenic NDV differentially modulate the expression of these receptors. Whereas the velogenic strains significantly downregulates the expression of CD69, B-Lec and IFN-γ of CD3^−^/28-4^+^ IEL-NK cells in chickens, infection with the lentogenic pathotype lead to the upregulation of the these receptors. Notably, the CD69 and IFN gamma are crucial for T cell proliferation (28) and NK cytotoxicity (29). Indeed, enhanced cytotoxicity of NK cells against some targets has been shown to be associated with increased expression of CD69 (29). Similarly, downregulation of CD69 expression by human herpes virus-6 was shown to significantly block the activation of T cells (30). In the current study, we also observed a close relationship between the reduction of NK cells and downregulation of CD69, IFN gamma, and B-Lec genes. Since both CD69 and B-Lec receptors are known to trigger NK cell cytotoxicity (31), and that increased expression of interferon gamma is positively correlated with NK cell functions, downregulating the expression of these receptors by velogenic NDV (Table 4) definitely leads to devastating clinical consequences in the infected chickens (Table 4). Further research is needed to functionally associate the expression profiles of these receptors with the virulence potential of NDV.

According to the “missing self” hypothesis, normal cells are prevented from the antiviral activity of NK cells through a constitutive expression of MHC class I molecules which are recognized by the inhibitory NK cell receptors. Down-modulation of the MHC-I due to virus infection, therefore, renders the infected cells vulnerable to NK cell killing. On the other hand, upregulation of MHC-1 on the infected cells increases the activity of the inhibitory NK receptors and, therefore, the inhibition of NK cytolysis. Therefore, by expressing ligands for inhibitory receptors on the surface of the infected cells, or increasing the expression of the inhibitory NK receptors, as exhibited by some viruses, the NK cell-mediated destruction of the virus-infected cells can be avoided. Human immunodeficiency virus (HIV) for instance, has been shown to outsmart NK cells by engaging the killer inhibitory receptors on the NK cells *via* its p24 epitopes and prevent the activation of NK cells (32). Similarly, murine cytomegalovirus prevents the activation of NK cells by encoding an MHC class I like molecule which serves as a ligand for some inhibitory receptors (33). In the current study, we showed that velogenic NDV upregulated the expression of B-NK, a potent inhibitory receptor found on the surface of NK cells. On the other hand, the lentogenic NDV only slightly upregulated the expression of this inhibitory gene (Table 4). Since the reduced activity of this receptor correlates with increased activity of NK cells, the enhanced pathogenicity of the velogenic NDV in chicken might not be unconnected with its ability to upregulate this receptor and in turn suppress the activity of the IEL-NK cells.

To further demonstrate the susceptibility of CD3^−^/28-4^+^ IEL-NK cells to NDV, we examined the cells using transmission electron microscopy. Our findings revealed the presence of virus-like particles within the cytoplasm of the CD3^−^/28-4^+^ IEL-NK cells infected with velogenic NDV (Figures 2B,C). The uninfected control (Figure 2A) and lentogenic NDV infected cells (Figure 2D), however, showed no clear intracytoplasmic localization of these virus particles. Moreover, the TEM results are consistent with the virus load determination based on the quantification of viral mRNA transcripts which showed an increase in the quantity of velogenic but not lentogenic NDV as the infection progressed (Table 2). It is, therefore, possible that velogenic but not lentogenic NDV, can infect and replicate in the avian CD3^−^/28-4^+^ IEL-NK cells thereby compromising the inherent antiviral mechanisms of the cells. Interestingly, many viruses including Epstein–Barr virus (34) and HIV (35) have been previously shown to infect the NK cells directly, trigger apoptosis, and interfere with the immunological roles of the cells.

Indeed, following infection with velogenic NDV, we observed a generalized cytoskeletal disfiguration of the CD3^−^/28-4^+^ IEL-NK cells characterized by irregular distribution of organelles. In particular, there was a marked increase in the number of mitochondria compared with the lentogenic and uninfected control. Such increased mitochondrial mass has been previously demonstrated in HIV-specific CD8^+^ T cells (36), cytomegalovirus-infected human embryonic fibroblasts (37) as well as the glioblastoma cells infected with enterovirus 71 (38). Obviously, the mitochondrion is the powerhouse of the cell responsible for the supply of energy needed for the maintenance of cell homeostasis (39). They are, therefore, involved in the regulation of many processes in the cell such as metabolism, cell cycle, signal transduction, and antimicrobial defense (40, 41). Consequently, for them to efficiently replicate, most viruses cause the loss of structural and functional integrity of the mitochondria leading to severe compromise of most of the cellular processes. As a compensatory mechanism, however, the mitochondria tend to undergo an adaptive response by increasing their numbers to meet up with the rising demand of energy in the cell. This may explain the unusual increase in mitochondria organelles observed in this study following velogenic NDV infection (Figures 2B,C).

Taken together, we have been able to isolate the CD3^−^/28-4^+^ IEL-NK cells from the pool of avian duodenal IELs and characterized them during NDV infection. We demonstrated the differential modulation of CD3^−^/28-4^+^ IEL-NK cells following velogenic and lentogenic infection. In particular, we showed that the interaction of the virulent NDV with the CD3^−^/28-4^+^ IEL-NK cells leads to the downregulation of activating receptors and the corresponding upregulation of the inhibitory NK cell’s receptor. We also showed a drastic reduction of CD3^−^/28-4^+^ IEL-NK cells following velogenic NDV infection compared to lentogenic NDV infection in chicken. Finally, we also provided the evidence that velogenic NDV but not the lentogenic strain, may infect, damage, and replicate within CD3^−^/28-4^+^ IEL-NK cells found in the chicken gut. To the best of our knowledge, this is the first study on the characterization of the IEL-NK cells surface receptors following velogenic and lentogenic NDV infection. More studies are required to determine the functional importance of our findings in this study.

## Ethics Statement

The animal challenge protocol was carried out in accordance with the recommendation of the Institutional of Animal Care and Use Committee (IACUC), Faculty of Veterinary Medicine, UPM (reference number UPM/IACUC/AUP-R067/2014); the local animal care authority. The animals used for this research were kept in the house and animal care procedures were conducted according to the local animal welfare regulations and EU directive (EU Directive on the protection of animals used for scientific purposes 2010/63/EU) (42) under bio-safety level (BSL2) enhanced experimental animal facility at the Faculty of Veterinary Medicine, UPM. Animals were monitored for a minimum of two times per day by a qualified and registered veterinarian to ensure animal welfare and health. The end point of all these *in vivo* animal experiments was death; although, a humane endpoint was pre-defined in the protocol and applied to prevent any pain, distress, or suffering. The humane endpoint was decided for animals manifested terminal clinical signs. Animals showing terminal signs including anorexia and paralysis were sacrificed by cervical dislocation under sedation in accordance with standard guidelines (42).

## Author Contributions

Conceived and designed the experiments: AI, AO, MA, MHB, ST, and SY. Performed the experiments: MA. Analyzed the data: MA, AO, DS, and MJ. Contributed reagents/materials/analysis tools: AI and AO. Wrote the manuscript: AO, MA, and MBB. Critical revision: AI and AO.

## Conflict of Interest Statement

The authors declare that the research was conducted in the absence of any commercial or financial relationships that could be construed as a potential conflict of interest.
