# Supplementary material for: Effects of Newcastle Disease Virus Infection on Chicken Intestinal Intraepithelial Natural Killer Cells
Source: Front Immunol. 2018 Jun 20;9:1386. doi: 10.3389/fimmu.2018.01386 (PMC6019501; doi:10.3389/fimmu.2018.01386)
Supplement: Supplementary file 1 [file Table_1.DOCX]

Supplementary Material

# Effects of Newcastle Disease Virus Infection on Chicken Intestinal Intraepithelial Natural Killer Cells

# Mostafa Abdolmaleki^1^, Swee Keong Yeap^1,3^, Sheau Wei Tan^1^, Dilan A. Satharasinghe^1^, Muhammad Bashir Bello^1^, Mohammad Zareian Jahromi^1^, Mohd Hair Bejo^1,2^, Abdul Rahman Omar^1,2^, Aini Ideris ^1,2*^

***corresponding author**

**Table S1|** Probes and primers sequences of real-time PCR assay.

**Housekeeping Genes**

| Genes |  | Primer and Probe Sequences(5^/^-3^/^) | Annealing Temperature $\boldsymbol{℃}$ | Accession Number | Reference |
| --- | --- | --- | --- | --- | --- |
| GAPDH | Probe^a^ F^b^  R^b^ | (FAM)-CGCCATCACTATCTTCCAGG-(BHQ1) GAACGGGAAACTTGTGAT GACTCCACAACATACTCA | 58 | NM 204305 | 17 |
| 18S | Probe  F  R | (FAM)-CCACAGTTA-ZEN-TCCAAGTAACGGGAGGG(IBFQ) TCAGTTATGGTTCCTTTGGTCG CGTCGGCATGTATTAGCTCTAG | 60 | AF173612.1 | This study |
| Beta Actin | Probe  F  R | (FAM)-ACCTTCAAC-ZEN-ACCCCAGCCATGTAT(IBFQ) ACCCCAAAGCCAACAGAG CCAGAGTCCATCACAATACCAG | 60 | L08165.1 | This study |

^a^FAM, 6-carboxyfluorescein; BHQ1, Black hole quencher; IBFQ, Iowa Black Fluorescent Quencher, ^b^F, forward primer, R, reverse primer

**NK cells related Genes**

| Genes |  | Primer and Probe Sequences(5^/^-3^/^) | Annealing Temperature $\boldsymbol{℃}$ | Accession Number | Sequence/  Reference |
| --- | --- | --- | --- | --- | --- |
| B-NK | Probe^a^  F^b^  R^b^ | - | 60 | NM-001044682 | ACGGGCAGTTTGCCTCC |
| NK-LYSIN | Probe  F  R | CCGGTCCCATTGCGTC TGCGTGGGATGCAGATGAAG CAGAATCTGCATTTAATCCCCTTGC | 60 | NM-001044680 | This study |
| CD69 | Probe  F  R | - | 60 | NM-0010807 | ACACGGAGGAGGAGAT |
| CHIR-AB1 | Probe  F  R | CCTTGGCACCTGTCAGC CCACTGTGGACATCAAATCAGAGT CCGCCACCAGCAGGTT | 60 | NM-0011461 | This study |
| B-Lectin | Probe  F  R | TTGCAGTGCAGGCATT TGTTCACAGTGCTGCTCATCA CAATCCAGTCGAAGGGACACT | 60 | AJ-634334 | This study |
| IFN-$\boldsymbol{\gamma}$ | Probe  F  R | (FAM)-TGGCCAAGCTCCCGATGAACGA-(BHQ1) GTGAAGAAGGTGAAAGATATCATGGA GCTTTGCGCTGGATTCTCA | 58 | Y07922 | 16 |

^a^FAM, 6-carboxyfluorescein; BHQ1, Black hole quencher; IBFQ, Iowa Black Fluorescent Quencher
 ^b^F, forward primer ; R, reverse primer
